# Urban Scaling and Its Deviations: Revealing the Structure of Wealth, Innovation and Crime across Cities

**DOI:** 10.1371/journal.pone.0013541

**Published:** 2010-11-10

**Authors:** Luís M. A. Bettencourt, José Lobo, Deborah Strumsky, Geoffrey B. West

**Affiliations:** 1 Theoretical Division and Center for Nonlinear Studies (CNLS), Los Alamos National Laboratory, Los Alamos, New Mexico, United States of America; 2 Santa Fe Institute, Santa Fe, New Mexico, United States of America; 3 School of Human Evolution and Social Change and W. P. Carey School of Business, Arizona State University, Tempe, Arizona, United States of America; 4 Department of Geography and Earth Sciences, University of North Carolina at Charlotte, Charlotte, North Carolina, United States of America; Universidade de Vigo, Spain

## Abstract

With urban population increasing dramatically worldwide, cities are playing an increasingly critical role in human societies and the sustainability of the planet. An obstacle to effective policy is the lack of meaningful urban metrics based on a quantitative understanding of cities. Typically, linear per capita indicators are used to characterize and rank cities. However, these implicitly ignore the fundamental role of nonlinear agglomeration integral to the life history of cities. As such, per capita indicators conflate general nonlinear effects, common to all cities, with local dynamics, specific to each city, failing to provide direct measures of the impact of local events and policy. Agglomeration nonlinearities are explicitly manifested by the superlinear power law scaling of most urban socioeconomic indicators with population size, all with similar exponents (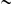
1.15). As a result larger cities are disproportionally the centers of innovation, wealth and crime, all to approximately the same degree. We use these general urban laws to develop new urban metrics that disentangle dynamics at different scales and provide true measures of local urban performance. New rankings of cities and a novel and simpler perspective on urban systems emerge. We find that local urban dynamics display long-term memory, so cities under or outperforming their size expectation maintain such (dis)advantage for decades. Spatiotemporal correlation analyses reveal a novel functional taxonomy of U.S. metropolitan areas that is generally not organized geographically but based instead on common local economic models, innovation strategies and patterns of crime.

## Introduction

How rich, creative or safe can we expect a city to be? How can we establish which cities are the most creative, the most violent, or most effective at generating wealth? The conventional answer is to use the rank order of per capita measures of performance [1], [2]. However, per capita indicators conflate general effects of urbanization, common to all cities as a function of their population size, with local events and dynamics that are specific to particular places. Because it is often the latter that are of most interest for scientific analyses that can inform policy decisions it is important to define a set of urban metrics of local performance that are independent of expectations due solely to population size.

Per capita measures of urban performance are ubiquitous in official statistics, policy documents and in the scientific literature. For example, official statistics on wages, income or gross domestic product (GDP) compiled by governmental agencies and international bodies worldwide [3] report on both total amounts and per capita quantities as a means to compare the economic performance of various places. Similarly, official crime statistics (see e.g. the FBI Uniform Crime Reports [4]) are expressed in terms of crime rates (number of crimes per 100,000 inhabitants per year). Many other important indicators that measure local economic and social well-being, such as unemployment rates, innovation rates (see e.g. [5]), cost of living index, morbidity and mortality rates, poverty rates, etc, all are reported on a per capita basis. Even well known composite indices of urban performance and quality of life, such as those compiled by *Fortune*, *Forbes* and *The Economist*, rely primarily on linear combinations of per capita quantities.

The use of per capita indicators assumes implicitly that, on average, specific urban characteristics, 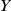, increase linearly with population size 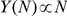. However, this approach is unsuitable for characterizing and comparing cities because it ignores the fundamental emergent phenomenon of agglomeration [6]–[11] resulting from non-linear interactions in social dynamics [6], [7], [10] and organization [11], [12] as cities grow. Such non-linearities are fundamental to the very existence of cities [6], [7], [9], [11], [13] and are manifested as systematic scaling laws [14]–[19] which explicitly show that cities are more than the linear sum of their individual components. For example, economic productivity [12], [13], [15], [20]–[22] (value-added in manufacturing, GDP, wages, personal income, etc.) increases systematically on a per capita basis by 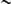
15% with every doubling of a city's population, regardless of a city's initial size (whether from, say, 50,000 to 100,000 or, from 5,000,000 to 10,000,000). Remarkably, these general increasing returns to population size manifest, on average, the same statistical relationship (the 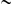
15% rule) across an extraordinarily broad range of metrics, regardless of nation or time. Similar increases apply to almost every socioeconomic quantity, from innovation rates [10], [14] and rhythms of human behavior [15] to incidence of crime [15], [16] and infectious diseases [15], [18]. They express a continuous and systematic acceleration of socioeconomic processes with increasing numbers of people [15], so that larger cities produce and spend wealth faster, create new ideas more frequently and suffer from greater incidence of crime all approximately to the same degree.

These empirical regularities strongly suggest that underlying these apparently diverse phenomena there is a universal socioeconomic dynamic reflecting average organizational behavior of human interactions in cities [15], [17]. From this perspective a city's population size is an aggregate proxy for a set of general processes facilitated by the co-location of many different individuals and social organizations, with different motivations and expertise. Such effects rely on more intense and effective social interactions as city size increases and have been described at length in sociology and economics [23]–[26]. Big cities derive many advantages from larger populations such as more efficient economic specialization and division of labor, more efficient socioeconomic matching that facilitates social and economic markets, easier sharing of resources resulting in greater economies of scale and faster learning and innovation from the observation and recombination of a larger and more diverse set of technological and organizational processes [27]. However, the difficulty persists that many of these processes, such as the idea of knowledge spillovers promoting innovation and economic growth [28], have remained very hard to quantify and model in general terms. As a consequence the relative importance of different detailed micro-level processes remains unclear and a subject of intense investigation in several disciplines [29]. It is in the aggregate of the city that these stochastic micro-processes add up to population size dependent stable averages, expressing the general effects of urbanization in terms of non-trivial scaling laws as functions of population size [15].

Thus, scaling laws provide the average baseline behavior and, by extension, the null model necessary for addressing the long-standing problem of how to rank specific cities meaningfully and assess the effects of local events, historical contingency, and policy, independently of population size. These agglomeration laws provide the *expected* average characteristics that a city of a given size should manifest in the absence of any specific local features. However, it is very often local characteristics, represented by how particular cities deviate from their expected baseline behavior, that are the most interesting for both policy and scientific analyses. Here, we show how deviations from scaling laws can be used to construct truly local measures of a city's organization and dynamics. As a result, we are able to address several fundamental questions such as how exceptional can a city be relative to its peers, what timescales are relevant for local policy to take effect, what are the local relationships between quantities such as economic development, crime and innovation, and whether each city is unique, or if there are identifiable (geographic) organizing principles expressed as shared patterns of urban development across families of cities.

## Results

Agglomeration effects in cities are typically manifested as (*i*) economies of scale in material infrastructure [11], [15], [17] (for example, as decreases in the per capita area of road surface or length of electrical cabling with increasing population size) and (*ii*) as increases in per capita temporal rates [15] of socioeconomic activities, such as wealth creation, innovation and crime. We focus on wealth creation, innovation and crime because they share a common origin in social interactions in cities and are key indicators of well-being. However, it should be kept in mind that the procedure described below applies equally well to any other urban indicator that scales systematically with population size.

**Figure 1:**
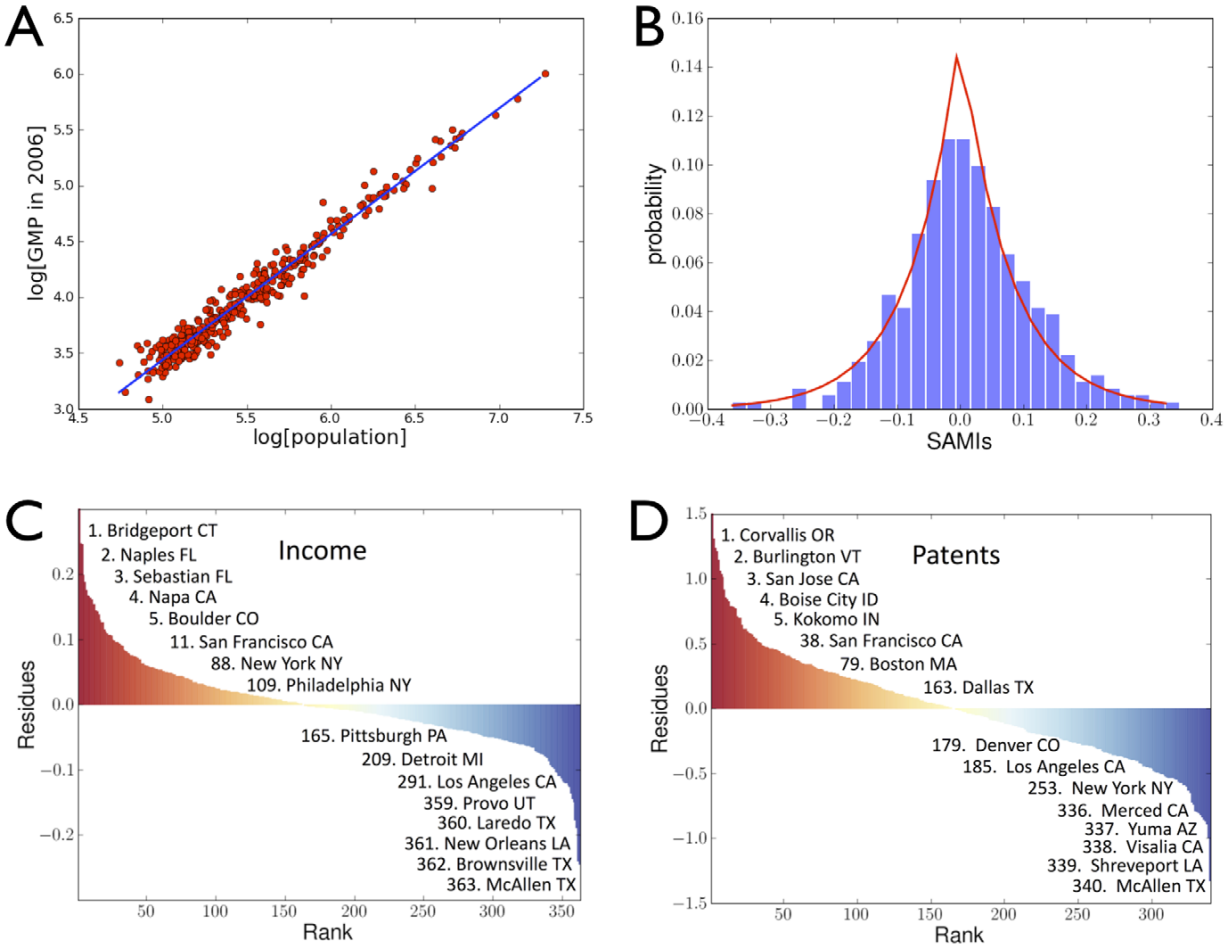
Urban Agglomeration effects result in per capita nonlinear scaling of urban metrics. Subtracting these effects produces a truly local measure of urban dynamics and a reference scale for ranking cities. a) A typical superlinear scaling law (solid line): Gross Metropolitan Product of US MSAs in 2006 (red dots) vs. population; the slope of the solid line has exponent, 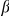 = 1.126 (95% CI [1.101,1.149]). b) Histogram showing frequency of residuals, (SAMIs, see Eq. (2)); the statistics of residuals is well described by a Laplace distribution (red line). Scale independent ranking (SAMIs) for US MSAs by c) personal income and d) patenting (red denotes above average performance, blue below). For more details see Text S1, Table S1 and Figure S1.

Non-linear agglomeration effects are manifested as simple scaling laws. Recent studies [14]–[19] have shown that most urban quantities, 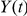, follow approximate power-law scaling
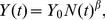
(1)where 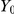 is a normalization constant, 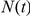 is the population size at time, 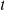, and 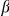 is the scaling exponent. Consequently, with each fractional increase of population size, 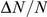, the relative increase in the per capita quantity, 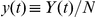, is, from Eq. (1), given by 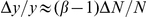. When 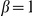, then, on average, 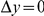, 
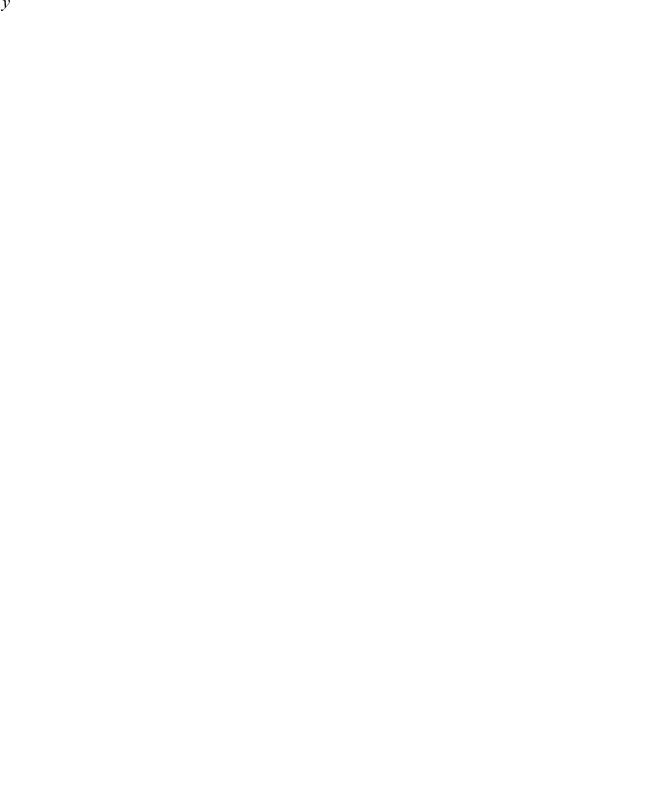
 is constant and 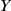 is linear in 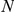. In this case, a standard per capita measure is the appropriate baseline for ranking deviations. However, for almost all quantities of interest 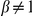 and the baseline itself is a function of 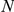. For material infrastructural quantities the exponent is sublinear, 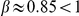, so that 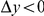, expressing economies of scale, whereas for socioeconomic quantities it is superlinear, 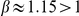, so that 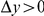, expressing increasing returns to scale. A typical example of an urban scaling law (Gross Metropolitan Product, or GMP) is shown in Figure 1A.

Eq. (1) is motivated by the more general observation that diverse characteristics of many complex adaptive systems, and especially those of biological organisms [30] and social systems with much in common with cities, obey simple nonlinear scaling laws. Furthermore, such systems often manifest a universal nonlinear behavior. In biology this is reflected in the predominance of approximate quarter-power exponents, whose origins are physical and geometric properties of underlying resource and information distribution network structures [17], [30] (e.g., vascular and neural systems). Similar scale-free, fractal-like behavior has been observed in many human social networks [31], including cities [17], [31]–[33]. It is therefore natural and compelling that the essential features of a quantitative, predictive theory of cities originate in the dynamics and form of social [34], [35] and infrastructural networks [11], [15], [33] and that these underlie the observed scaling and the approximate universal values of the exponents, 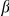.

For a given value of 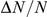, 
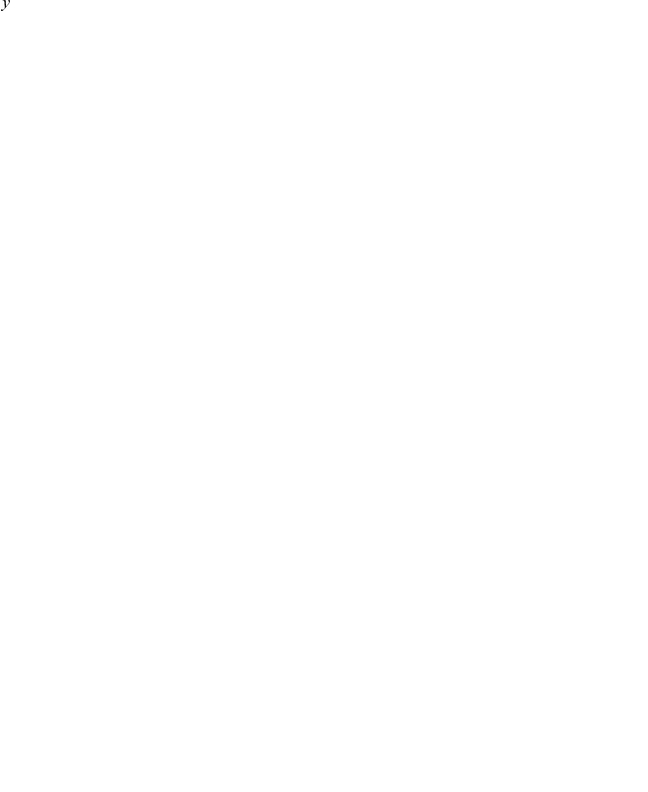
 depends only on 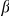 but not on initial city size, 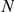, expressing the principle that a meaningful comparison between cities should rely on *relative* quantities rather than on their absolute values. Eq. (1) is analogous to a mean-field description and expresses the average behavior of urban metrics, 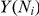, for a city of population 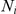. Deviations from this average (the analogues to statistical fluctuations) parametrize the characteristics of each individual city. These are quantified by the residuals [36],
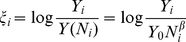
(2)where 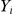 is the observed value of the metric for each specific city. We refer to the 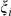 as *Scale-Adjusted Metropolitan Indicators* (SAMIs). Unlike per capita indicators, SAMIs are dimensionless, independent of city size (Figure 1B) and usually of other urban metrics such as land area or population density (see Discussion and Text S2). SAMIs capture human and social dynamics specific to a given place and time - its true local flavor - and represent its successes or failures relative to other cities. They allow direct comparison between any two cities and provide meaningful rankings across the urban system.

To illustrate this methodology and its potential impact we analyze data from U.S. Metropolitan Statistical Areas (MSAs) (see Materials for data sources and city definitions). These are socioeconomic units defined via commuting flows, in contrast to more arbitrary political divisions such as counties or administrative cities. This definition emphasizes social interactions as the defining feature of cities. It attempts to circumscribe the city geographically as a mixing population where all residents can come into contact with each other, a familiar concept in epidemiology and ecology [37].

We find that the variation in local quantities corresponding to different cities in the same year is well characterized statistically by a Laplace (exponential) distribution density
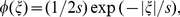
(3)where 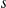 parametrizes its width, or more precisely the mean expectation for the absolute value of SAMIs 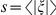. Figure 1B shows the normalized SAMI histogram (the estimate of the SAMI probability density function) for 360 MSAs, in good agreement with the prediction from the Laplace distribution (red line).

Interestingly, this Laplace distribution for SAMIs implies that the statistics of the urban indicators themselves also follow a power-law distribution density. Substituting, the definition of SAMIs, Eq. (2), into the Laplace distribution (3), and accounting for the change in measure in the probability density 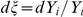, allows us to derive the statistics of the original indicators 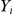 as
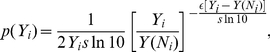
(4)where the number 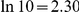 and the sign function 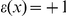 for 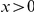 and 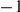 for 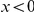. The average value of 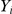 is given by the scaling law Eq. (1). The average magnitude of the deviations from scaling, namely the width of 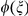, 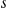, depends on the given quantity, but is stable over long periods of time (for instance, decades for personal income and patents). Its values are larger for patents (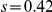) than for violent crime (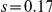), and significantly larger than for economic quantities, such as income (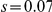) or GMP (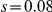). Thus, these economic quantities are least sensitive to local variation with 93–96% of their variance being predicted solely by population size (see Table S1). Violent crime follows, where scaling accounts for 86% of the variance while patents are subject to stronger local factors, having a wider SAMI distribution, with scaling effects accounting for about 65–70% of the variance in the data.

The first use of SAMIs is to provide a meaningful way to rank cities. Figure 1C,D show two examples of rankings of approximately 360 US MSAs in 2005 by the magnitude and sign of their SAMIs for income and patents. Complete tables are available online (http://www.santafe.edu/urban_observatory/). Compared to per capita indicators, which place 7 of the largest 20 MSAs in the top 20 for GMP, SAMIs show no population size bias, ranking none of these cities in the top 20. SAMIs also reveal that New York is quite an average city, marginally richer than its size might predict (rank 88th in income, 184th in GMP), not very inventive (178th in patents) and quite safe (267th in violent crime). San Francisco is the most exceptional large city, being rich (11th in income), creative (19th in patents) and fairly safe (181th in violent crime). The truly exceptional MSAs are smaller, such as Bridgeport for income, Corvallis and San Jose (Silicon Valley) for patents and Logan or Bangor for safety.

**Figure 2:**
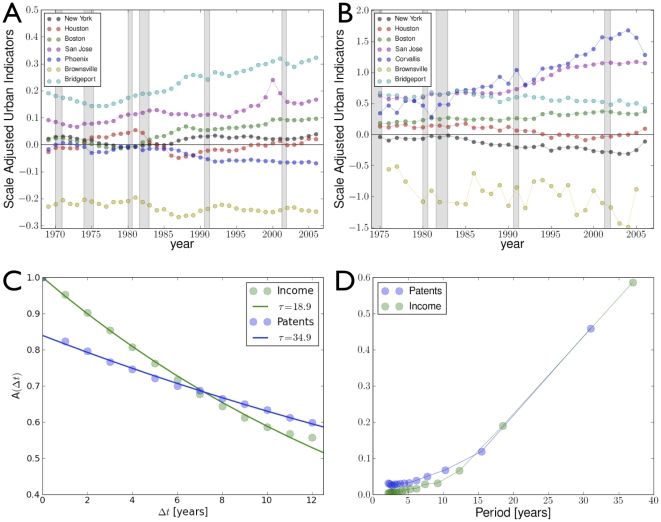
The temporal evolution of scale independent indicators (SAMIs) displays long-term memory. The value of SAMIs as functions of time for a) income (1969–2006) and b) patents (1975–2006) for selected MAs. Shaded grey areas indicate periods of national economic recession. The temporal autocorrelation c) for patents and personal income and exponential fits, 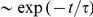, with characteristic decay times of 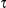 = 18.9 and 34.9 years, respectively and d) temporal Fourier power spectrum for the same quantities shows that their dynamics is dominated by long timescales.

**Figure 3:**
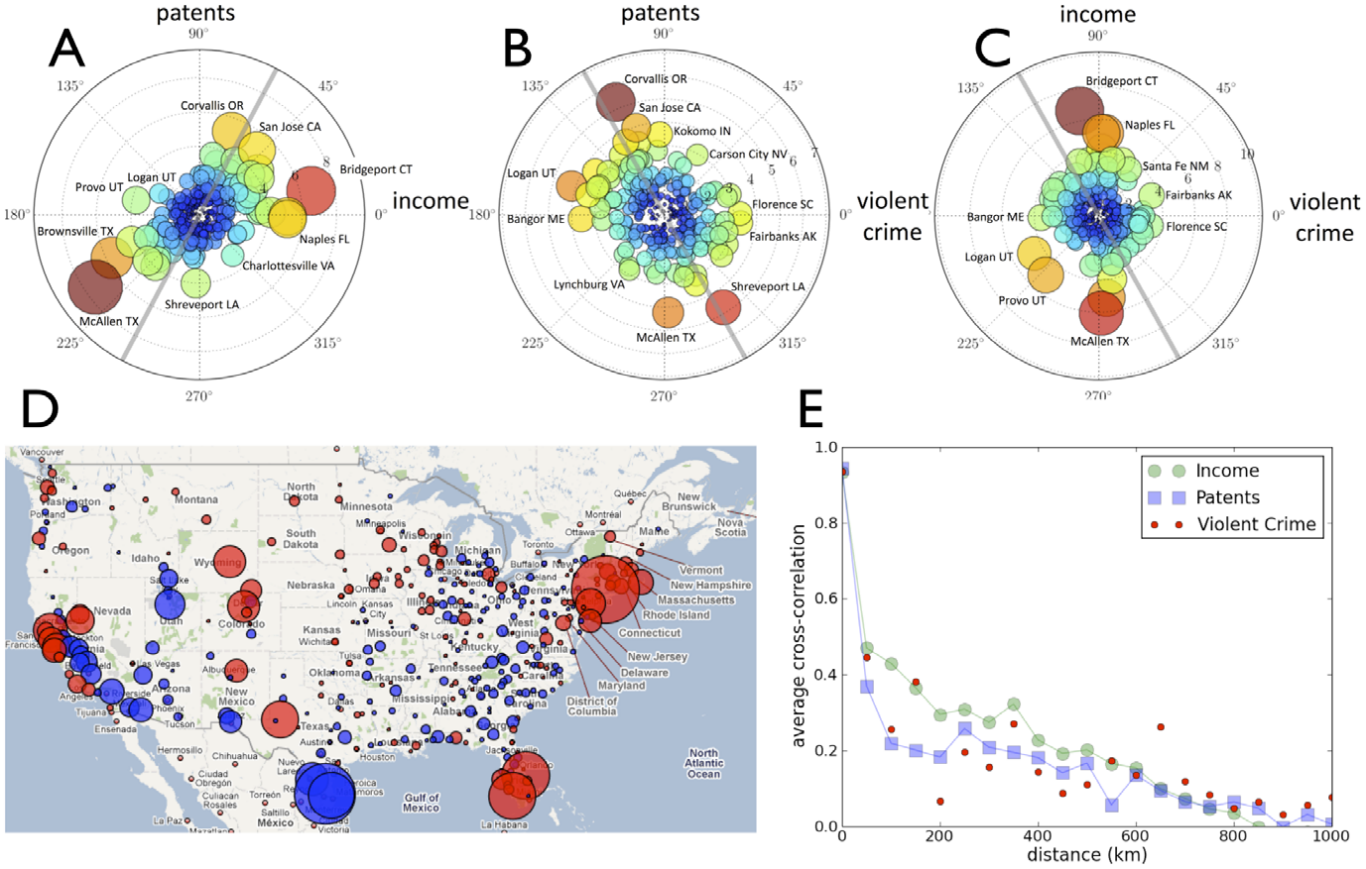
Relationships between local urban performance measured by personal income, patents and violent crime and their spatial distributions. A) normalized SAMIs for income versus patents are shown in polar coordinates, see SI, together with best-fit linear relation capturing overall average correlation (solid line, gradient = 0.38
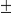
0.04, 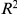 = 0.20). The color and size of circles both denote the magnitude of the combined SAMIs for each city; b) similar representation for income versus violent crime with best-fit linear relation (gradient = −0.19
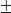
0.07, 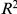 = 0.05), and c) similar representation for patents versus violent crime with best-fit linear relation (gradient = −0.34
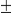
0.05, 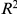 = 0.12). Note that B) and C) show a small amount of anti-correlation between SAMIs, which contrasts with the positive correlations for the per capita quantities due to their size dependence. d) Spatial distribution of income residuals (SAMIs) in 2006 (created with Google maps, see online (http://www.santafe.edu/urban_observatory/).). Red (blue) dots correspond to deviations above (below) expectation for city size. The size of the circle denotes the magnitude of the SAMIs. e) Average cross-correlation between SAMIs versus spatial separation distance, showing short-range spatial correlation. Averages shown are subject to large variation for distances 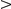
200 km (124 miles) with standard deviation 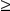
0.6.

**Figure 4:**
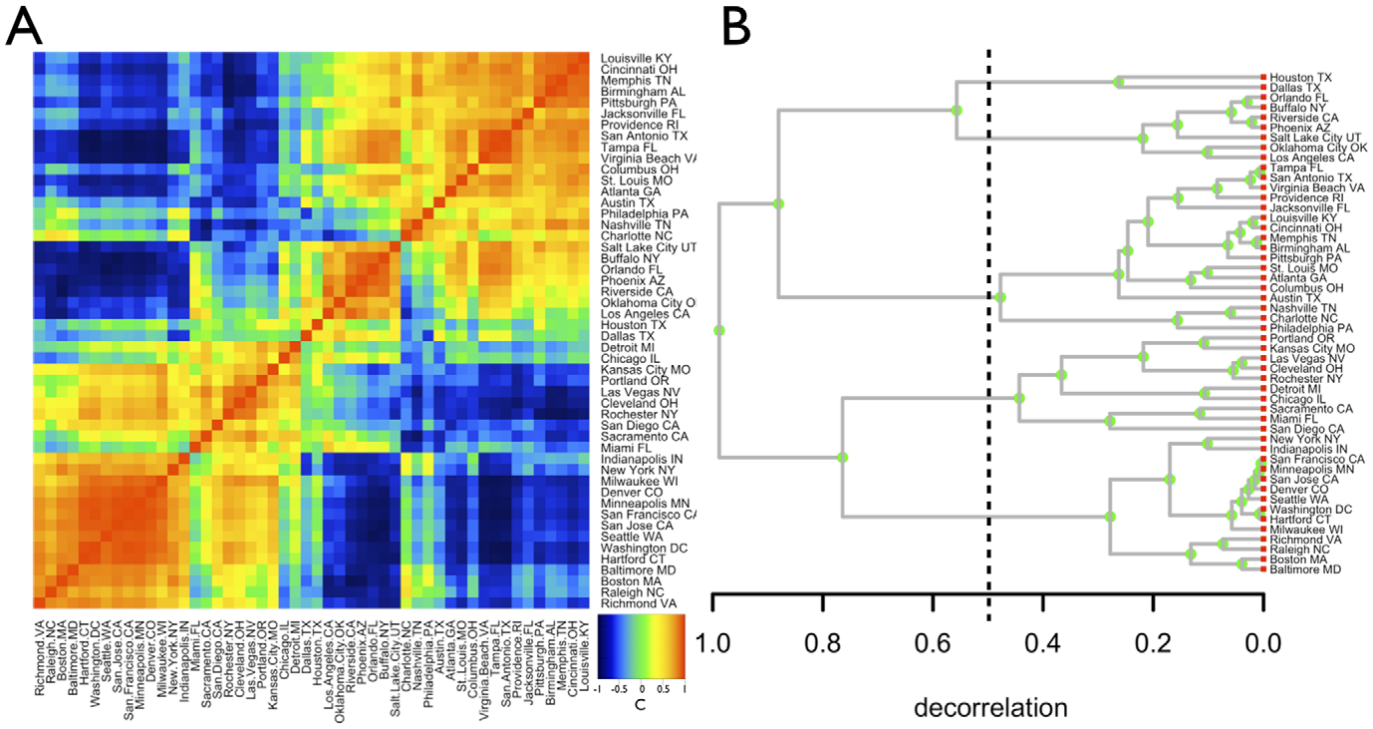
Families of kindred cities. The cross-correlation between SAMI time-series gives a measure of similarity, which can be used to group cities into clusters with similar characteristics; A) sorted correlation matrix (heatmap) for personal income in US MSAs with population over 1 million. Red (blue) denotes greatest (dis)similarity; B)Dendrogram showing detailed urban taxonomy of USMAs according to personal income. This clearly manifests clustering among cities with similar time trajectories. Here we used a decorrelation measure 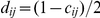 as distance between any two cities, where 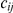 is the cross-correlation of Figure 4A. When the decorrelation 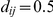, 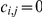, indicating no correlation(dashed line), revealing five families of kindred cities. See Figures S2, S3, S4, S5, S6, and S7 for other quantities.

The probability distribution of SAMIs, Eq. (3), might suggest that they behave much like random fluctuations. However, as illustrated in Figures 2, 3, and 4, they display strong regularities both in time and between cities. For instance, Figure 2A and B show the temporal trajectory of SAMI values for a few typical cities for personal income and patents over nearly four decades. The persistence in time of SAMIs indicates that even as cities gain or loose population, local characteristics are preserved and, in many instances, are reinforced to a surprising degree. Thus, the most salient feature of Figure 2A,B is how slow fundamental urban change actually is [38]. Most cities that were rich and innovative in the 1960s tend to remain rich today, and rankings of poor and technologically disadvantaged cities likewise persist over the same period. The change in a city's performance is measured by the auto-correlation of its metrics over time, 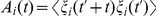 (see Materials and Methods), and by the Fourier temporal power spectrum 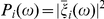 of urban trajectories (see Methods for definitions). Their averages over all cities for personal income and patents are illustrated in Figure 2C and D, respectively, and show that, although there is change on short time-scales, most dynamics happens over characteristic time-scales of decades.

In general, higher rates of violent crime positively correlate with higher average incomes. However, this is primarily because both quantities scale similarly with city population size. SAMIs allow us to factor out these dominant general size effects and identify local relationships. Figure 3A, B, C show polar plots of these quantities in 2005. These representations are obtained by dividing the SAMIs for each quantity by 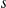, and plotting the result in coordinates on the circle. Although SAMIs have different average magnitudes, 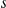, for different quantities this representation allows us to show exceptional cities in terms of two variables at once on the same scale. Once population size effects are removed, the remaining correlations between income, patents and personal crime are, in fact, weak. However, some trends persist (solid lines), showing that cities that outperform in income tend to outperform in patents, and that underperformance in both is positively correlated with higher rates of violent crime [39]. There are some notable exceptions, however, as shown in Figure 3. In this sense, it is possible to be poor but safe (Logan, Provo) or rich and violent (Fairbanks, Santa Fe).

Place and geography are important in the development of cities [11], [12], [40], [41], so that it is interesting to investigate if urban performance of nearby cities is similar. Figure 3D shows the spatial distribution of SAMIs for personal income (see online (http://www.santafe.edu/urban_observatory/). for more maps and years). Such maps reveal regions with clusters of cities that under or over-perform relative to size expectations, resulting in short-distance positive correlations between local dynamics. However, these effects average out among neighboring cities with different characteristics so that significant average spatial correlations disappear for distances 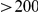 km (Figure 3E). Consequently, spatial proximity is not in general a good determinant of similarity.

This lack of greater spatial similarity in socioeconomic SAMIs raises the question of whether the local dynamics of different cities are idiosyncratic and unique (random spatial fluctuations), or whether there are common patterns across the urban system. To investigate this question we ask more specifically if the SAMI histories of different cities, see Figure 2A, B, are similar for the same indicator. We measure similarity by the equal time cross-correlations of SAMIs time-series (see Methods for definitions). Once computed we can use this measure of similarity as a distance with which to cluster cities into classes of urban dynamics. Cities showing the highest cross-correlations not only perform similarly relative to expectation for their size, but also have similar local histories. This suggests the concept of *kindred cities* exhibiting common characteristic advantages and challenges as they evolve. These clusters of similar cities can be visualized using a heatmap, which is a correlation matrix sorted by similarity, grouping together sets of similar MSAs (denoted in red) and separating anti-correlated ones (blue); see Figure 4A for personal income for US MSAs above 1 million. An equivalent representation is the taxonomic tree shown in Figure 4B (for patents, violent crime and GDP see Figures S2, S3, S4, S5, S6, and S7). At any given level of similarity this tree can be cut into a number of non-overlapping families of kindred cities. When, as is natural, we choose as the benchmark the point where the value of the cross-correlation vanishes and two cities are neither positively nor negatively correlated, we obtain only five families of cities. These clusters do not generally correspond to geographic proximity, but reflect instead commonalities of economic choices and historical paths. Examples include the cluster containing San Francisco, San Jose, Minneapolis, Denver and Seattle as high-tech centers, and Pittsburgh, Cincinnati, Memphis and Birmingham as market and transportation hubs with industrial pasts.

## Discussion

In this paper, we have proposed a systematic procedure for solving the long-standing problem of constructing meaningful, science-based metrics for ranking and assessing local features of cities [39]. By using nonlinear urban scaling laws as a baseline, our procedure accounts for the underlying principles and socioeconomic dynamics that give rise to cities to distinguish general effects of urbanism from local dynamics and, consequently, leads to a much simpler and direct perspective into the local factors that make or break specific places.

Population size plays a fundamental role in this approach. In the spirit of the successful application of scaling analysis to many other system - from collective physical phenomena [42] to biological organisms and ecosystems [30], [43]–[46] - the systematic variation of the properties of cities with population size reveals the ways in which cities result in more than the simple agglomerations of people. This is the phenomenon that anthropologist Carneiro described as *quality from quantity* in his studies of the emergence of organizational forms in small human societies [47]. Scaling laws for cities show systematic effects of spatial densification, temporal acceleration and socioeconomic diversification, that have long been discussed in the social sciences [23]–[26], but that can only now start be appreciated for their quantitative generality. In particular two general aspects of the scaling properties of urban indicators appears systematically across time, and in different urban systems: i) economies of scale in urban material infrastructure and ii) increasing returns in socioeconomic productivity. Whenever these two general effects can overcome other socioeconomic disruptions, such as expensive transportation and social insecurity, cities become magnets for human social activity. Thus, population size is not so much a causal force, but rather a proxy aggregate variable that denotes a set of diverse socio-economic mechanisms that derive advantages from the co-location and intense interaction of people. The general regularity of urban scaling laws and of the statistics of their deviations point to the possibility of a general theory of cities that can account for the essence of these interactions and predict a small set of fundamental scaling regularities common to all urban systems.

From this viewpoint, the general statistically stable properties of cities emerge as a hierarchy of interrelated fundamental quantities. First, it has been known for some time that the population size distribution of cities has remained relatively stable over time and across many different nations and is well-described by a Zipf power law distribution [48], [49]. Analogously, we have shown [15] that scaling laws for socioeconomic and infrastructural metrics persist over time and across every nation that has been studied, and that these organize urban quantities into two broad universality classes of dynamics that manifest either increasing returns to scale (socioeconomic quantities) or economies of scale (material infrastructure) both to approximately the same degree. Here, we have taken the analysis a step further and shown that the deviations from these generic scaling laws, which express local factors specific to individual cities, also manifest distributions and correlations that are surprisingly stable over long times. These distributions represent averages over much faster individual and social dynamical processes, including changes in personal behavior, social contact structure, and migration. It is therefore extraordinary that, despite the immense diversity of human and social behavior, the dynamics and organization of urban systems, as well as of individual cities, is an emergent predictable phenomenon.

Secondly, perhaps the most conspicuous property of SAMIs is that they do not randomly fluctuate over time but, instead, show long temporal persistence. This indicates that, even though the size and structure of a city's population may change considerably over time, any initial advantage or disadvantage that it has relative to its scaling expectation tends to be preserved over decades. In this sense, either for good or for bad, cities are remarkably robust. Examples are Phoenix, which has remained a mild economic under-performer over the last four decades maintaining a similar value of 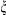 for personal income even as the city nearly quadrupled in population since the late 1960s (Figure 2A). Or, the initial advantages of San Jose (Silicon Valley) in terms of wealth creation and innovation which was already present in the 1960s. This over-performance was sustained and even reinforced over forty years, despite the short term boom and bust technological and economic cycle in 1999–2000, at the end of which the city returned to its long term basal trend (Figure 2A). Put slightly differently: apart from a relatively small bump in the late 1990s, the continued success of San Jose was already set well *before* the birth of Silicon Valley. Other examples that deal with population loss are also illuminating. Former industrial cities, such as Pittsburgh or Buffalo, have now experienced almost four decades of slow population loss, despite massive interventions to reverse such trends. The recent histories of these cities are also characterized by negative SAMIs (especially for income) and by their lowest levels coinciding with the greatest population loss. These examples suggest that, at least in part, we should think of cities as sets of socio-economic processes with a temporal persistence much longer than that of typical policy initiatives or the participation of particular individuals. In this sense, urban policy that promotes population growth as a means to benefit from the effects of agglomeration leaves the character of a city, including most of its challenges, unresolved, and may, in fact, contribute to exacerbate them. Policies that focus instead on establishing beneficial fundamental change in local urban dynamics will be very difficult to achieve but very much worth creating, as they will position a city for a long run of prosperity and innovation. It would be interesting to investigate whether similar long term memory and persistence of urban dynamics is also a property of fast changing urban systems such as those in China or India. We intend to explore some of these important questions in future work.

Our analyses show that average spatial correlations between cities in the US are relatively short ranged (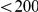 km) and may have been weakening over time. Thus, compared to their temporal persistence, geographic proximity is, at best, a weak predictor of the characteristics of a city. This is perhaps surprising in view of classical models of urban settlement and growth [11], [40], [41], which assume a close interdependence of a city and its surrounding area. The present lack of greater close spatial similarity may be the result of elevated and increasing mobility within the US [50], so it would be interesting to analyze urban systems in other nations where these effects may have played out differently.

Despite the lack of greater similarity due to geographical proximity, we find that most cities in the US show strong similarity with groups of other cities so that all US MSAs fall into a small number of classes of kindred cities sharing common historical paths. The same is true in terms of dissimilarity (or negative correlation) among cities, indicating that beneficial periods in specific sectors of the urban system coincide with negative developments in others, as Figure 4A illustrates. In fact, it is particularly interesting and perhaps surprising that these classes of local urban dynamics are not more diverse but, instead, fall into just a few groups, as quantified by local urban trajectories for personal income, patents and violent crime. The non-local nature of the similarity among urban trajectories strongly suggests that policy-makers should not search for analogous challenges and solutions in nearby cities but should instead consider who their kindred cities are.

Finally, it is important to emphasize that the average properties of most socioeconomic quantities such as wealth creation, crime and innovation are strongly predicted by the scaling laws expressed in Eq. (1), which are non-linear functions of population size and account for 65–97% of the variance in the data (see Table S1). The shape of the city in space, including for example its residential density, matter much less than (and are mostly accounted for by) population size in predicting indicators of urban performance. Said more explicitly, whether a city looks more like New York or Boston or instead like Los Angeles or Atlanta has a vanishing effect in predicting its socio-economic performance. However, there are, of course, some specific urban quantities that depend additionally on other properties of the city such as its spatial layout or climate. Examples are energy spent on transportation or climate control, and related emissions of pollutants. In these cases analyses of local indicators (SAMIs) will show dependence on other general urban variables, such as population density or urban area, which, on average, do not affect the quantities studied here.

In summary, we have used the empirical manifestations of the underlying principles of agglomeration and the implicit network structures and dynamics responsible for the formation of cities to account systematically for urban dynamics at different scales. This paradigm allows us to separate measures of true local dynamics and organization in cities from their generic universal behavior. We have shown that these local indicators (SAMIs) have well defined statistics and that the consideration of their temporal and spatial properties is an essential element of models and theory of urban evolution and a new tool for the formulation of improved urban policy.

## Materials and Methods

### Data sets and sources

Our spatial unit of analysis is the metropolitan statistical area (MSA). MSAs are defined by the U.S. Office of Management and Budget and are standardized county-based areas having at least one urbanized area of 50,000 or more population, plus adjacent territory that has a high degree of social and economic integration with the core, as measured by commuting ties. Data on Gross Metropolitan Product (GMP) was recently made available by the US Department of Commerces Bureau of Economic Analysis and is a measure - in 2001 chain-weighted dollars - of the market value of final goods and services produced within a metropolitan area in a particular period of time. Data on the number of violent crimes is provided by the US Federal Bureau of Investigation (Uniform Crime Reports). Metropolitan patent counts were constructed using data provided by the U.S. Patent and Trademark Office, see Text S1. Data on personal income and population was obtained from the US Bureau of Economic Analysis Regional Economic Information System.

### Scaling analysis and residual statistics

Data for GMP, personal income, violent crime and patents for each MSA corresponding to the same year were transformed logarithmically and fitted using Ordinary Least Squares to the logarithm of population, according to (1). Residuals from these fits, 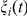, which we call Scale-Adjusted Metropolitan Indicators (SAMIs) were then isolated and binned to form a normalized histogram, from which a probability distribution is constructed. Both Gaussian and Laplacian (exponential) distribution functions were fitted to the resulting distribution using standard maximum likelihood estimators, see Table S1 The goodness of fit was evaluated in terms of the 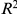 of these fits to the cumulative residuals distribution; see Figure S1.

### Urban ranking

The magnitude of the SAMIs corresponding to a given quantity and year for each city were used to rank cities. Two examples are shown in Figure 1A, B. Their spatial distribution are shown in Figure 3D, and online (http://www.santafe.edu/urban_observatory/).

### Temporal analysis

The temporal autocorrelation is defined as
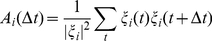
(5)where 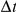 is measured in years. Dividing by 
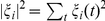
 ensures the normalization 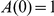. In practice, because the length of the vectors 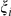 are finite in time we also take into account the multiplicities of the overlap relative to the equal time norm, so that 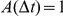, for 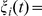
constant. The temporal autocorrelation was computed for each MSA using the corresponding time-series of personal income (period 1969–2006) and patents (1975–2005). Individual city autocorrelation functions were averaged to produce the points shown in Figure 2C. These data were then fitted to an exponential curve to obtain the characteristic decay times 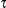. In Figure 2D the SAMI time-series for patents and personal income for each city were Fourier transformed and their power spectra produced as a function of temporal frequency. Individual power spectra were averaged over cities to produce the points shown.

### Polar plots

In Figure 3 A, B, C SAMIs were divided by their average distribution width 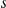 computed via fitting of a Laplace (exponential) distribution to the normalized histogram of residuals for a given year (see also Table S1). Resulting quantities were then combined two by two to produce polar plots, where, for each city (represented by a point in the polar diagram), the radius is the square root of the sum of the SAMI amplitudes for the two quantities and the polar angle is its phase.

### Interactive Online Maps

Interactive maps and tables of SAMIs for each quantity and year were produced using Exhibit (http://simile.mit.edu/wiki/Exhibit) and Google maps (http://maps.google.com). The figure shows one example. The full set can be viewed online (http://www.santafe.edu/urban_observatory/).

### Spatial autocorrelation

Spatial similarity between cities was computed in terms of the equal-time cross-correlation of their SAMI time-series
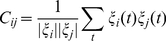
(6)This definition ensures that cities with similar values of SAMIs and time series (up to a multiplicative constant) will have the highest similarity. Distance was computed as the straight line connecting the two cities, by converting GIS coordinates to physical distance. Pairs of cities were grouped in distance bins of 50 km and averaged over all pairs to produce the points shown in Figure 3E. The standard deviation of these averages is large (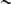
0.6 for pairs of cities beyond 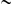
200 km). Thus, we find no significant average spatial correlations between local trajectories except for short distances.

### Urban similarity, clustering and heatmaps

Heatmaps were created by clustering the SAMI 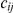 for pairs of cities. High cross correlation is shown in warm colors (red), while anti-correlation correlation is shown in cold ones (blue). The corresponding dendrogram groups cities together in terms their similarity. The measure of decorrelation shown is 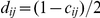, so two cities 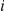 and 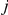 that are perfectly correlated have 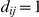, while cities that are maximally anti-correlated have 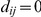; 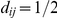 corresponds to 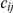 = 0, i.e. no correlation.

## Supporting Information

Figure S1 Fit of cumulative exponential (Laplace) and Gaussian distributions to residuals for personal income in 2005. Both distributions give an excellent fit, but the exponential (Laplace) distribution is better, especially for residues around zero.
(3.33 MB TIF)

Figure S2 Dendrogram of U.S. metropolitan areas grouped by incidence of violent crime, for cities with population above 1 million. Only cities reported by the FBI every year between 2001–06 are shown.
(1.92 MB TIF)

Figure S3 Heatmap of U.S. metropolitan areas grouped by incidence of violent crime for cities with population above 1 million. Only cities reported by the FBI every year from 2001 to 2006 are shown.
(11.97 MB TIF)

Figure S4 Dendrogram of U.S. metropolitan areas grouped by patenting rates for cities with population above 1 million. Data covers the period of 1975–2005.
(8.04 MB TIF)

Figure S5 Heatmap of U.S. metropolitan areas grouped by patenting rates for cities with population above 1 million. Data covers the period of 1975–2005.
(12.02 MB TIF)

Figure S6 Dendrogram of U.S. metropolitan areas grouped by Gross Metropolitan Product (GMP) for cities with population above 1 million. Data covers the period of 2001–2006.
(7.62 MB TIF)

Figure S7 Heatmap of U.S. metropolitan areas grouped by Gross Metropolitan Product (GMP) for cities with population above 1 million. Data covers the period of 2001–2006.
(2.94 MB TIF)

Table S1 Summary statistics for 2005. Scaling exponent with 95% confidence interval and R-squared for log-log fits of total urban indicator versus total population. Two fits to the residual distribution using an exponential (Laplace) and Gaussian distributions. The parameter s measures the width of the Laplace distribution. Similarly, σ is the standard deviation of the Gaussian. Values of R-squared shown for these parameters indicate goodness of fit of the cumulative residual distributions to the data (see Figure S1).
(0.05 MB DOCX)

Text S1 This describes in greater detail our methodology for assigning patents to metropolitan statistical areas.
(0.11 MB DOCX)

Text S2 This contains a summary of statistical analysis of correlations between SAMIs and population growth rates of Metropolitan Statistical Areas.
(0.08 MB DOCX)
