# Supplementary figures and images for: Urban Scaling and Its Deviations: Revealing the Structure of Wealth, Innovation and Crime across Cities
Source: PLoS One. 2010 Nov 10;5(11):e13541. doi: 10.1371/journal.pone.0013541 (PMC2978092; doi:10.1371/journal.pone.0013541)

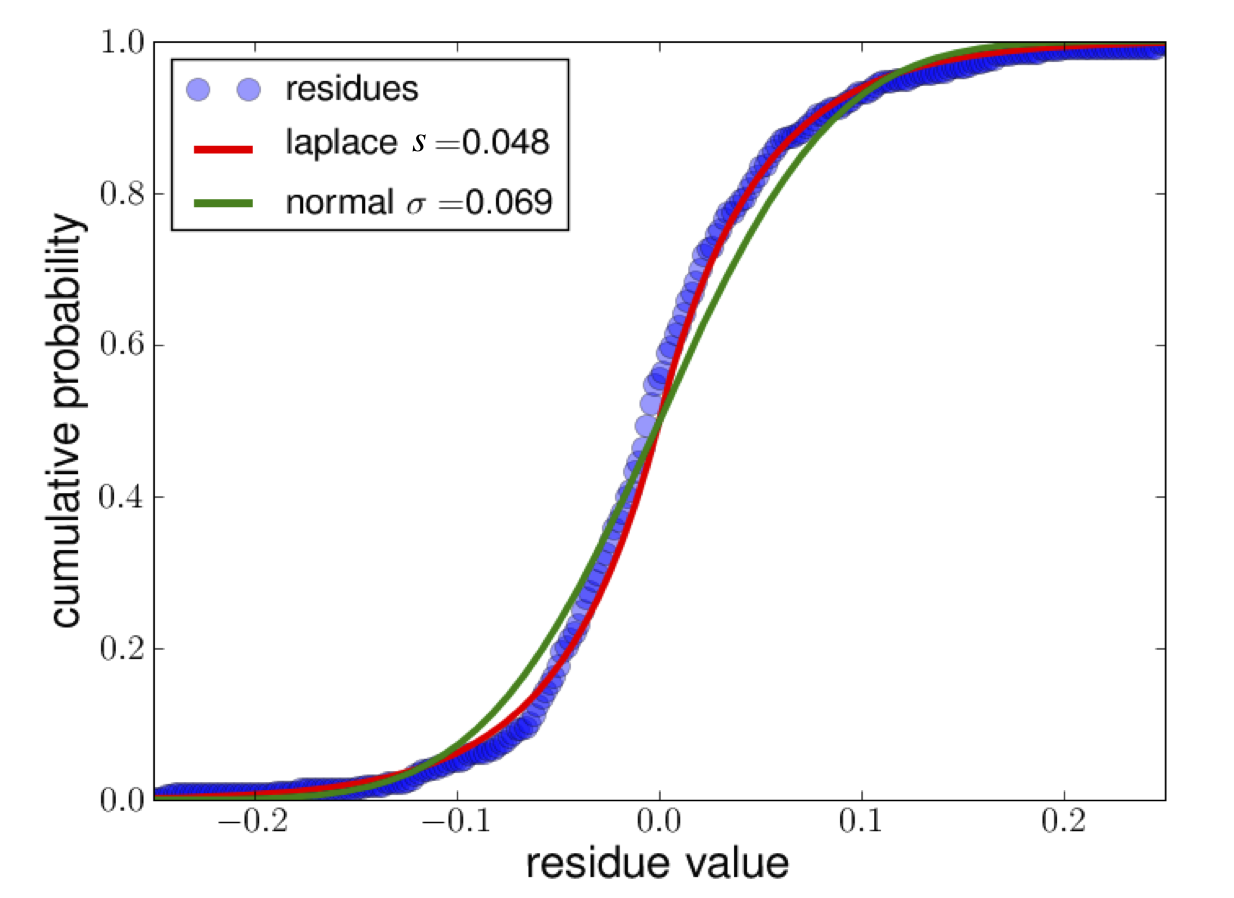

Supplement: Figure S1 — Fit of cumulative exponential (Laplace) and Gaussian distributions to residuals for personal income in 2005. Both distributions give an excellent fit, but the exponential (Laplace) distribution is better, especially for residues around zero. (3.33 MB TIF) [file pone.0013541.s001.tif]

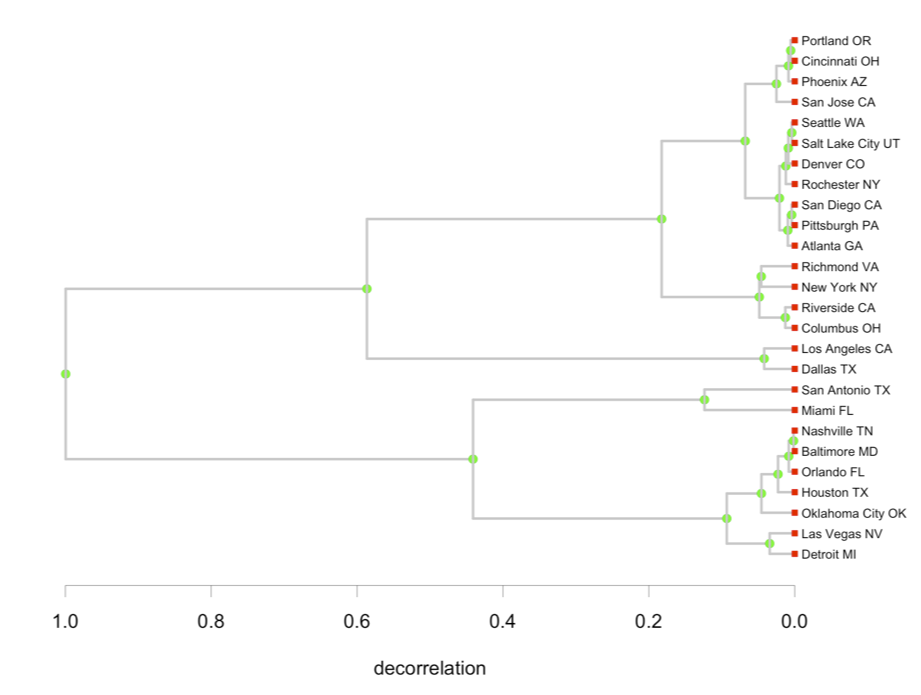

Supplement: Figure S2 — Dendrogram of U.S. metropolitan areas grouped by incidence of violent crime, for cities with population above 1 million. Only cities reported by the FBI every year between 2001–06 are shown. (1.92 MB TIF) [file pone.0013541.s002.tif]

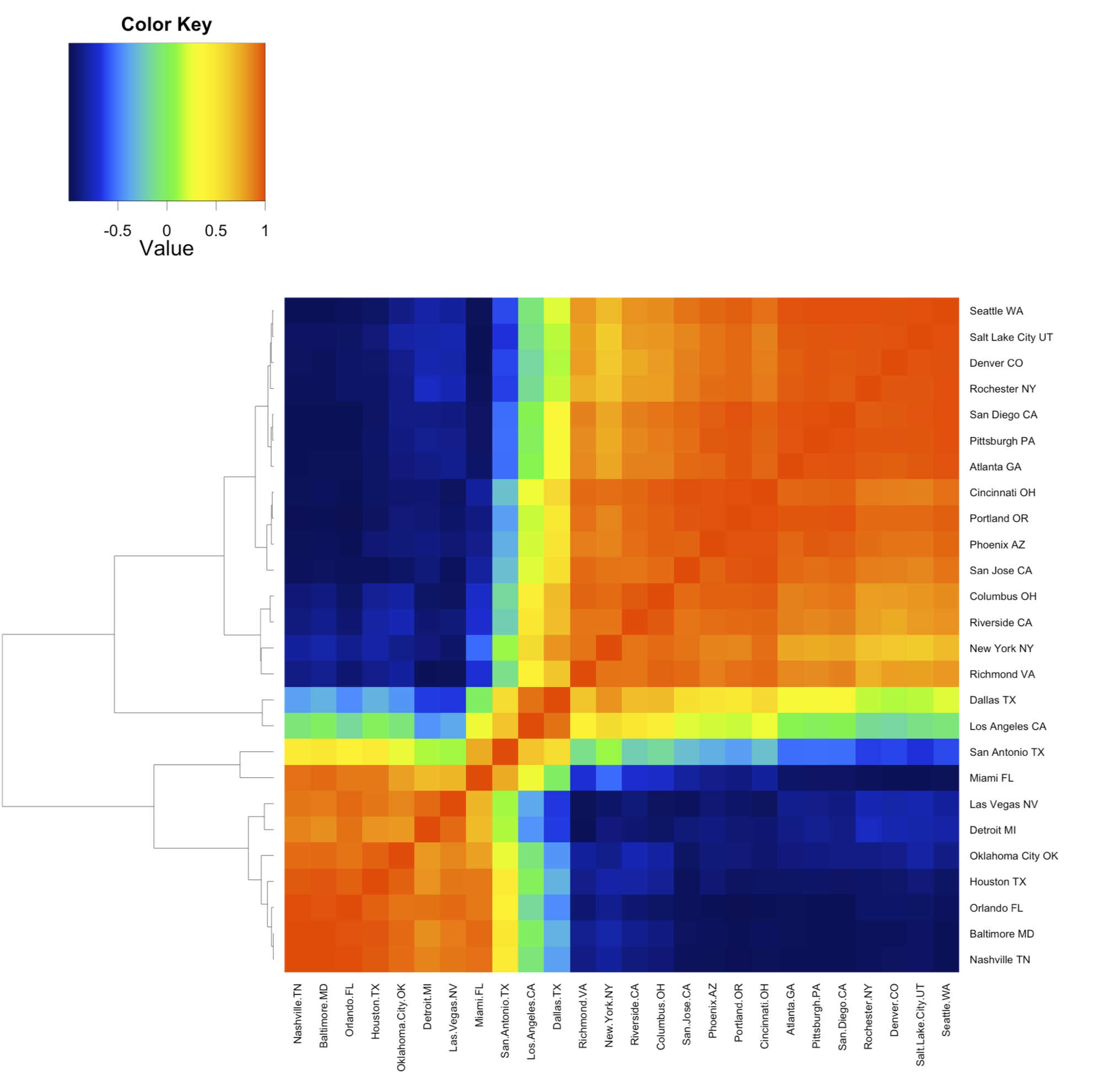

Supplement: Figure S3 — Heatmap of U.S. metropolitan areas grouped by incidence of violent crime for cities with population above 1 million. Only cities reported by the FBI every year from 2001 to 2006 are shown. (11.97 MB TIF) [file pone.0013541.s003.tif]

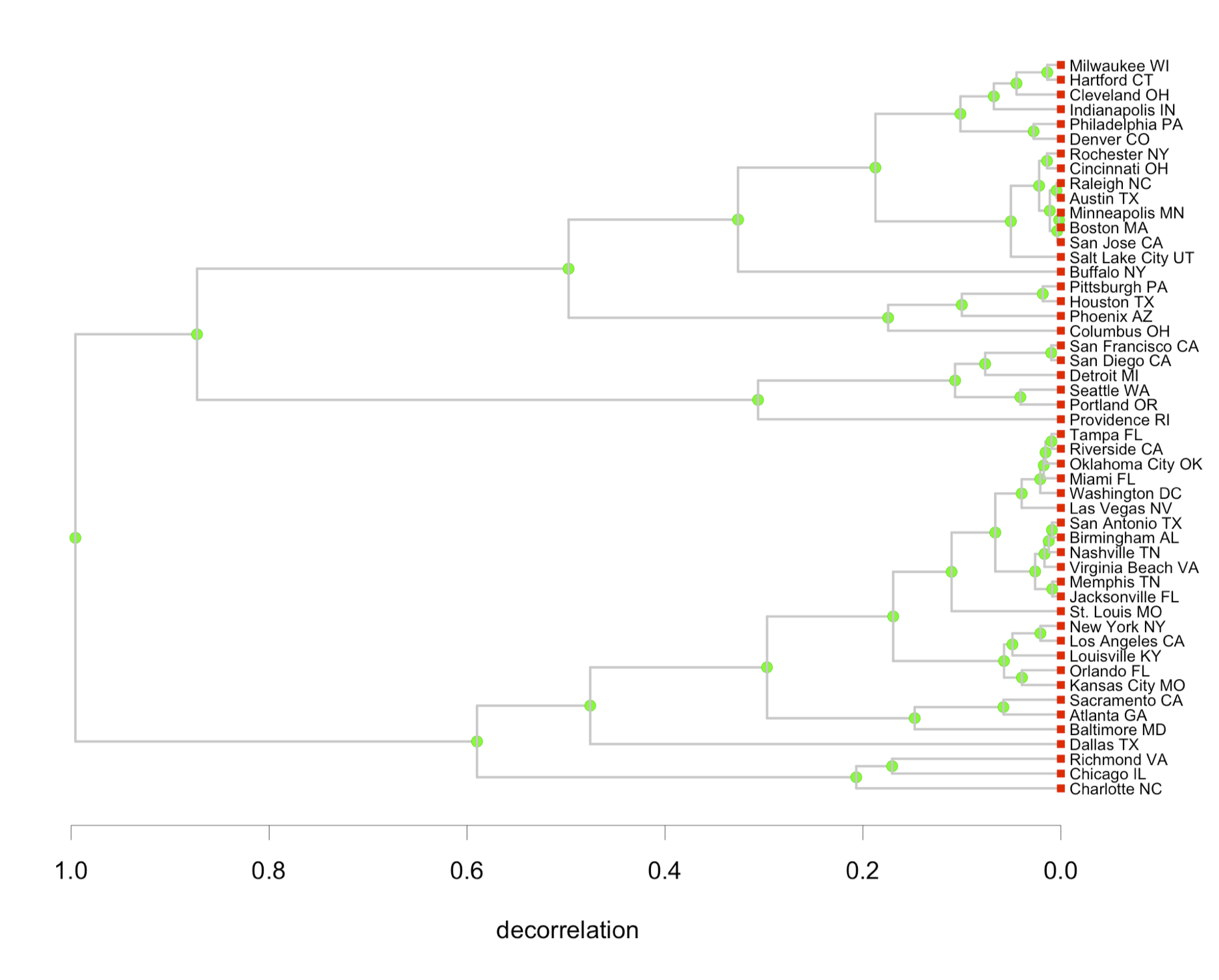

Supplement: Figure S4 — Dendrogram of U.S. metropolitan areas grouped by patenting rates for cities with population above 1 million. Data covers the period of 1975–2005. (8.04 MB TIF) [file pone.0013541.s004.tif]

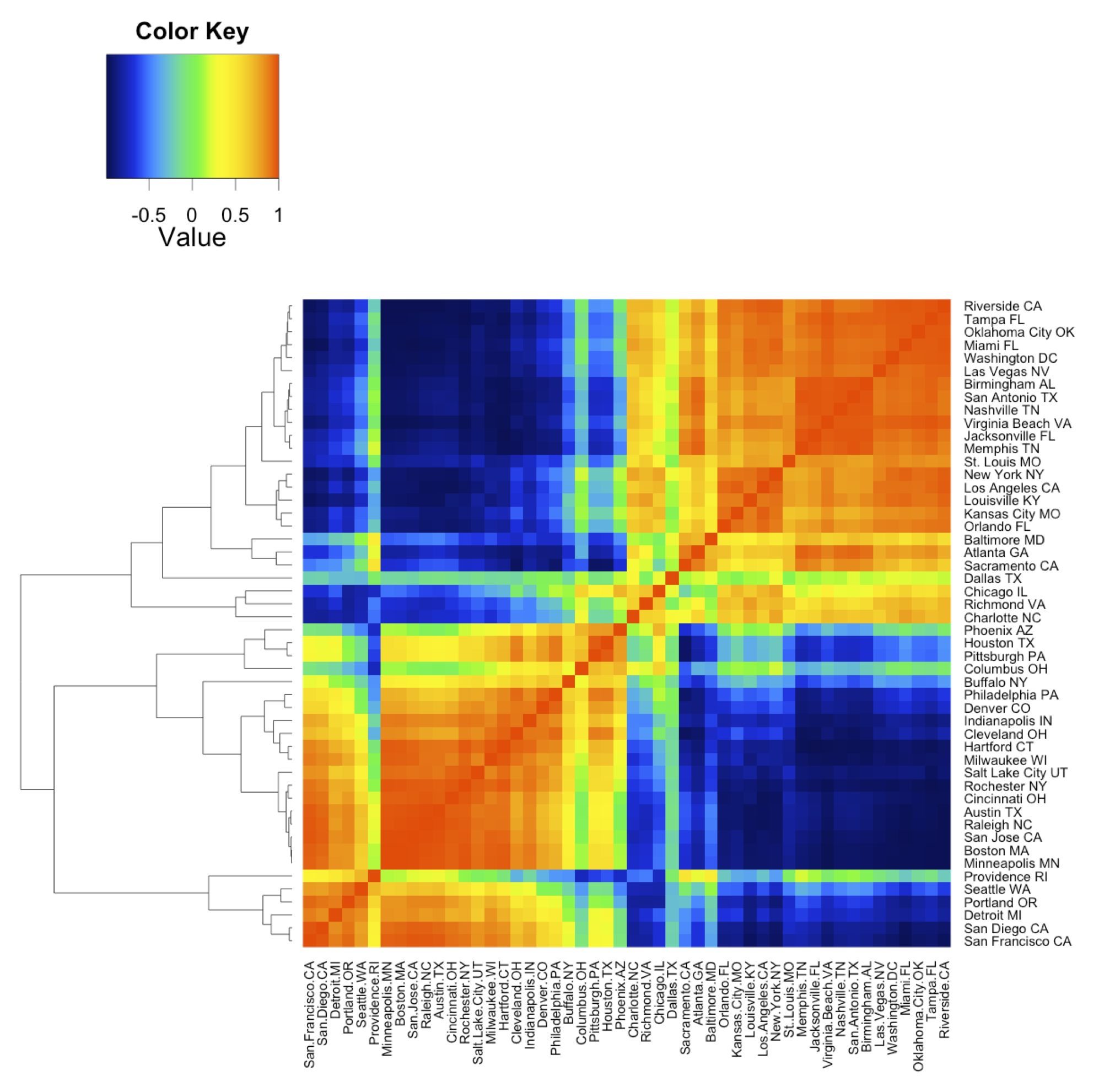

Supplement: Figure S5 — Heatmap of U.S. metropolitan areas grouped by patenting rates for cities with population above 1 million. Data covers the period of 1975–2005. (12.02 MB TIF) [file pone.0013541.s005.tif]

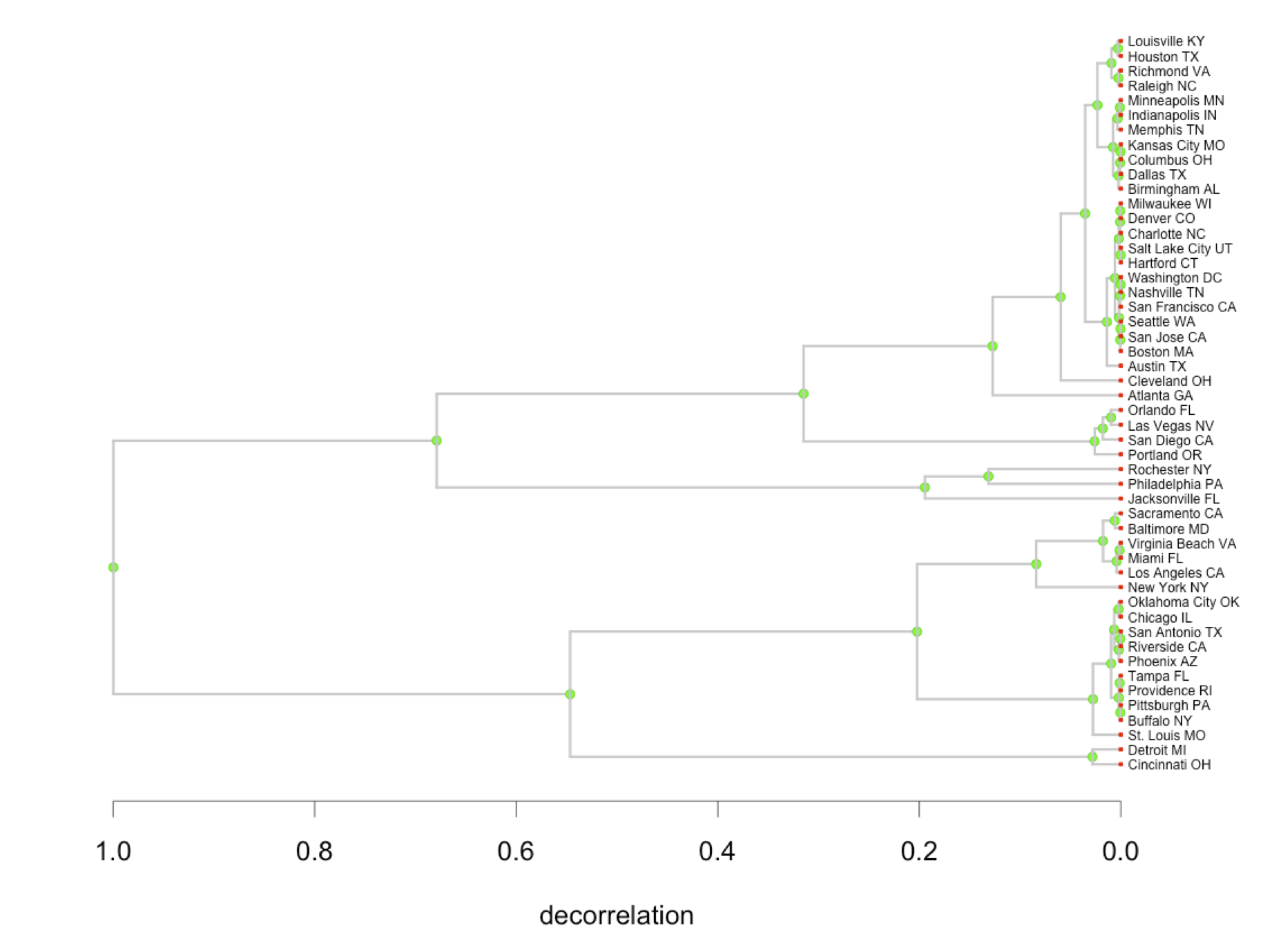

Supplement: Figure S6 — Dendrogram of U.S. metropolitan areas grouped by Gross Metropolitan Product (GMP) for cities with population above 1 million. Data covers the period of 2001–2006. (7.62 MB TIF) [file pone.0013541.s006.tif]

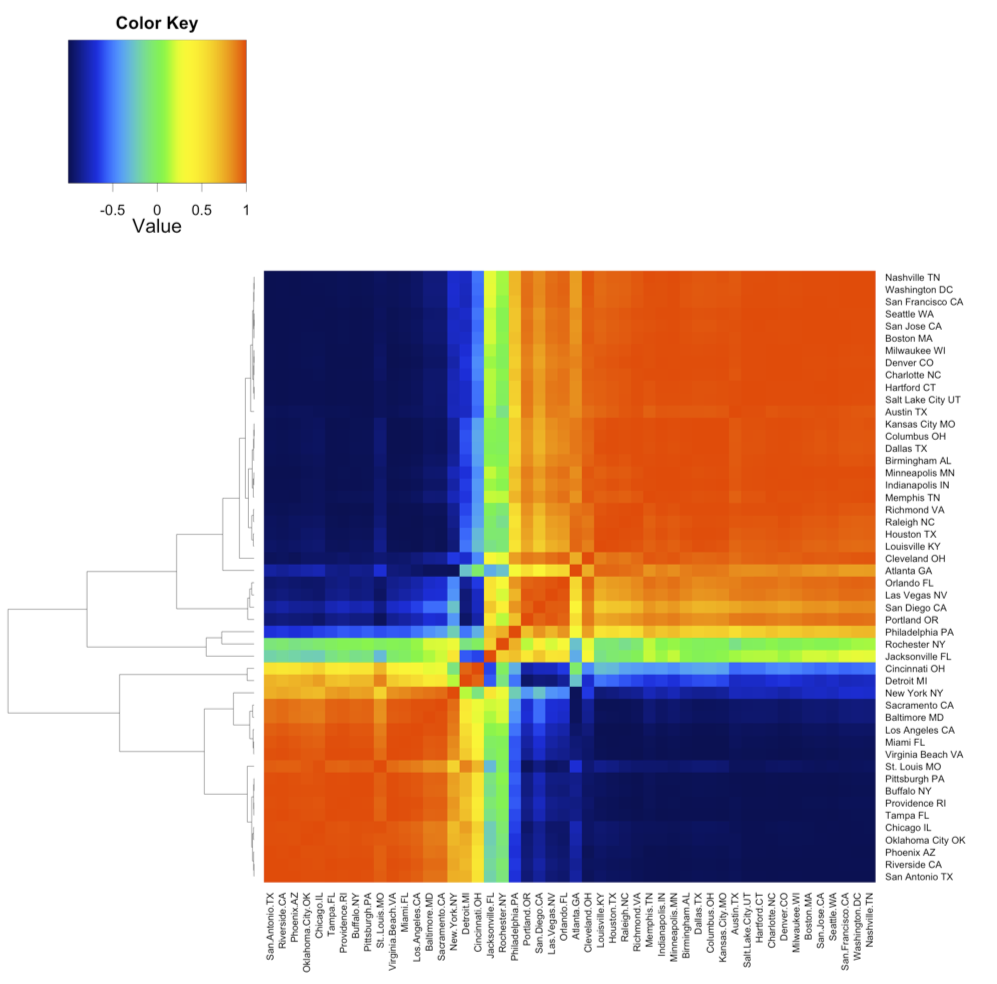

Supplement: Figure S7 — Heatmap of U.S. metropolitan areas grouped by Gross Metropolitan Product (GMP) for cities with population above 1 million. Data covers the period of 2001–2006. (2.94 MB TIF) [file pone.0013541.s007.tif]
