# Supplementary material for: Urban Scaling and Its Deviations: Revealing the Structure of Wealth, Innovation and Crime across Cities
Source: PLoS One. 2010 Nov 10;5(11):e13541. doi: 10.1371/journal.pone.0013541 (PMC2978092; doi:10.1371/journal.pone.0013541)
Supplement: Table S1 — Summary statistics for 2005. Scaling exponent with 95% confidence interval and R-squared for log-log fits of total urban indicator versus total population. Two fits to the residual distribution using an exponential (Laplace) and Gaussian distributions. The parameter s measures the width of the Laplace distribution. Similarly, σ is the standard deviation of the Gaussian. Values of R-squared shown for these parameters indicate goodness of fit of the cumulative residual distributions to the data (see Figure S1). (0.05 MB DOCX) [file pone.0013541.s008.docx]

|  | Scaling exponent  *b* | Residual distribution  s | Residual distribution  σ |
| --- | --- | --- | --- |
| Gross Metropolitan Product | 1.123 [1.101, 1.146]  (R^2^=0.929) | 0.077 (R^2^=0.998) | 0.100 (R^2^=0.998) |
| Personal Income | 1.082 [1.066,1.097] (R^2^=0.963) | 0.069 (R^2^=0.998) | 0.048 (R^2^=0.995) |
| Patents | 1.284 [1.181,1.386] (R^2^=0.665) | 0.424 (R^2^=0.997) | 0.330 (R^2^=0.998) |
| Violent Crime | 1.174 [1.123,1.225] (R^2^=0.864) | 0.171 (R^2^=0.996) | 0.215 (R^2^=0.999) |
