# Supplementary material for: Urban Scaling and Its Deviations: Revealing the Structure of Wealth, Innovation and Crime across Cities
Source: PLoS One. 2010 Nov 10;5(11):e13541. doi: 10.1371/journal.pone.0013541 (PMC2978092; doi:10.1371/journal.pone.0013541)
Supplement: Text S1 — This describes in greater detail our methodology for assigning patents to metropolitan statistical areas. (0.11 MB DOCX) [file pone.0013541.s009.docx]

**Patent Metropolitan Counts**

In order to obtain counts of metropolitan invention and inventors, source data was extracted from the U.S. Patent Office (USPTO) records on all granted patents. Every patent includes all inventors’ last names (with varying degrees of first and middle names or initials), each inventor’s home town, detailed information about the patent’s technology is defined through a set of well over 150,000 distinct codes, and the discrimination of the owner, or assignee, of the patent (generally a firm, and less often a university or government agency, if not owned by the inventor). Patent filings do not, however, provide consistent listings of inventor names or unique identifiers for the authors. Since the USPTO indexes source data by patent number and not by inventor, a variety of conditional matching algorithms were used to identify inventors, each inventor’s patents and other inventors with whom the focal inventor has co-authored at least one patent (the matching procedures are discussed in detail in [1]). Every inventor’s hometown was matched to a zip code, which was then assigned to an MSA using the ZIPList5 dataset. Ziplist5 is a commercially available dataset (www.zipinf.com) containing every active ZIP code currently defined by the U.S. Postal Service for the entire USA. Every zip code is assigned to an MSA if the zip code lies within a metropolitan county. The MSA county definitions used by ZIPList5 are consistent with the Census Bureau’s 2000 MSA definitions. Counties were assigned to MAs according to the MSA definitions used to create the metropolitan inventor networks. The analyses presented here relied upon all patents with at least one inventor within a metropolitan area. Thus, if two inventors from different metropolitan areas co-authored a patent, each metropolitan area’s count is increases by a weighted share (in this case 50% each). Patent co-authorship refers to the situation where a patent is either applied for by more than one individual or lists more than one individual as a designated inventor.

1.Marx, M., Strumsky, D., Fleming, L. Mobility, Skills, and the Michigan Non-Compete Experiment. *Management Science* **55**, 875-889 (2009).
