# Supplementary material for: Urban Scaling and Its Deviations: Revealing the Structure of Wealth, Innovation and Crime across Cities
Source: PLoS One. 2010 Nov 10;5(11):e13541. doi: 10.1371/journal.pone.0013541 (PMC2978092; doi:10.1371/journal.pone.0013541)
Supplement: Text S2 — This contains a summary of statistical analysis of correlations between SAMIs and population growth rates of Metropolitan Statistical Areas. (0.08 MB DOCX) [file pone.0013541.s010.docx]

**Correlation between residuals and metropolitan population growth and density**

We tested for correlations between a city’s residuals (SAMIs) and its population growth and population density. Population growth rate r is defined as r=ln[N(t+1)/N(t)], where N(t) is the population of a city (MSA) at time t, measured yearly. Population density was computed as the ratio of total metropolitan population to total area.

We might hypothesize that cities that are successful beyond their size in terms of income, patents or low incidence of crime may attract population at faster rates and thus show a positive correlation between these quantities and growth. Denser population may afford economies of scale in urban infrastructure that may increase the city’s ability to support a population. It has also been shown that denser urban areas provide improved opportunities to solve matching problems (finding research team partners).

However, no significant trend exists between residuals for income, patents and violent crime and population growth or density. Linear relations between these quantities resulted in fits with R^2^ consistent with zero (R^2^ < 0.1).

Among the fastest growing large cities in the USA Phoenix AZ and Las Vegas NV are not distinguished for high levels of income, innovation or for low incidence of crime. It has been argued that the relatively low price of real estate and the expectation of growth has played a role in attracting population to these areas.
